# Digital technology in medical visits: a critical review of its impact on doctor-patient communication

**DOI:** 10.3389/fpsyt.2023.1226225

**Published:** 2023-07-27

**Authors:** Filomena Marino, Francesca Alby, Cristina Zucchermaglio, Marilena Fatigante

**Affiliations:** Department of Social and Developmental Psychology, Sapienza University of Rome, Rome, Italy

**Keywords:** patient centered approach, digital technology, literacy practices, medical communication, medical visit

## Abstract

With the rapid advances of digital technology, computer-mediated medical practices are becoming increasingly dominant in medical visits. However, the question of how to ensure effective, patient-centered communication in this transition remains crucial. In this mini-review, we explore this topic by reviewing quantitative and survey-based studies, as well as discursive-interactional studies that focus on the visit as a communicative event. The review is organized into four sections: the introductory section provides a brief synthesis of the two main models used in medical practice and describes the effects of patient-centered communication practices on patients’ health and well-being. The second section presents and discusses qualitative and quantitative studies that assess the effect of technology on medical interaction and its impact on patient-centered communication. The third section focuses on whether and how the digital medical record represents a “potential communication risk” during doctor-patient interactions and explores how certain pen-and-paper literacy practices could help mitigate these challenges. In the concluding section, we outline and analyze three key considerations for utilizing technologies to foster and enhance patient-centered communication during medical visits.

## Introduction

The practice of medicine is guided by two basic approaches: the biomedical and biopsychosocial models. The biomedical model focuses solely on the biological aspects of disease (1), with the doctor as the only one possessing the technical and scientific knowledge to set the agenda (content and structure) of the visit (2). Criticism of the biomedical model has led to the development of the biopsychosocial approach. In this model, the patient’s experience (for example about his own lifeword) is considered relevant (3, 4). Both the doctor and patient contribute to the visit’s accomplishment by mobilizing their respective resources and competencies (5, 6). The biopsychosocial model promotes a patient-centered approach to medicine (7), emphasizing the importance of patient-centered communication (PCC). However, it is not uncommon for physicians to adopt a paternalistic communication style (8) despite recognizing the relevance of psychosocial aspects of care.

What are the main characteristics of patient-centered communication model? We can, according to the literature (9–15), identify four of them: (1) empathy: physicians demonstrate empathy towards patients, showing understanding and sensitivity to their experiences, emotions, and needs; (2) active involvement: PCC actively involves the patient in the decision-making process, allowing them to express their preferences and opinions regarding their care and engaging the patient as a partner in the care journey; (3) active listening: physicians dedicate time and energy to listen carefully to patients, allowing them to express their symptoms, concerns, and questions without interruptions or judgments; (4) clear and understandable communication: physicians use clear and understandable language to explain medical information and treatment options to patients, ensuring that patients can fully comprehend and participate in their care.

PCC encompasses co-constructed affective, participatory, and instrumental communicative behaviors that emerge in the interaction, taking into account the specific activity to be performed and the characteristics of each patient and illness. The accomplishment of the visit is constructed (and reconstructed) through discourse and constantly negotiated between doctor and patient. In medical consultations informed by a patient-centered communication model (PCC), active involvement of the patient is required (16, 17).

PCC has a positive impact on the care relationship, patients’ satisfaction, and well-being. It is associated with lower levels of emotional distress and anxiety, as well as improved quality of life (18, 19). Moreover, receiving clear and detailed information about their clinical condition and participating in treatment decisions reduces patients’ anxiety, improves their sleep quality, and increases their satisfaction and trust in their physician (20). Patients who are actively involved in clinical decision-making are more satisfied after the visit, have a better understanding of their disease, are better able to control their clinical condition, and experience a better quality of life after diagnosis and treatment (21, 22). Furthermore, doctor’s empathic listening, addressing patients’ doubts and fears, and reassuring patients contribute to establishing a trusting relationship, greater physician compliance, better psychological health, less emotional distress, and lower anxiety (23–25).

These impacts of PCC on health outcomes suggests that it is relevant to explore how it is affected by changes in technology-mediated medical practice. In the following, we will specifically analyze how the literature has described the relationship between the presence of technology and the patient-centered communication model. The aim of this review is to identify the conditions, resources, and constraints to implement patient-centered communication with patients, even in the face of the extensive and pervasive transition to digital technology in medicine.

## Patient-centered communication in a technology-driven medical landscape

Over the last 30 years, the use of computers and electronic medical records (EMRs) has become widespread in medicine. Across all medical specialties, doctors have transitioned from traditional paper and pen practices to incorporating or augmenting them with technology-mediated approaches in the management of medical visits. This change has enabled quick access to clinical information, easier management of drug prescriptions and more efficient retrieval and storage of medical records (26). Furthermore, computer usage has reduced medical errors by enabling easy access to scientific literature, healthcare guidelines, and drug composition (27–30).

In a recent literature analysis on the impact of technology on doctor-patient communication, Elkefi and Asan (31) highlight how the utilization of different technologies (such as patient portals, artificial intelligence, electronic health records, telemedicine) can empower cancer patients. This empowerment facilitates decision-making and supports their active engagement in the care processes, leading to improved health outcomes. Additionally, the use of technology contributes to maintaining a positive relationship between patients and physicians and enables the enactment of patient-centered communication.

However, some studies indicate that using computers during medical consultations can also have adverse effects on doctor-patient communication and relationships. For instance, physicians may find it challenging to divide their attention between the computer and the patient. Specifically, looking at the screen or typing on the computer may increase the risk of physicians not listening carefully and not answering the patient’s questions (32).

Ethnographic, observational and conversational studies of computer use during videotaped doctor-patient consultations emphasize that computers have a fundamental role in shaping the interaction during the visit. Pearce et al. (33) point out that the computer becomes an important communicative actor during medical consultations, which both physician and patient must take into account in managing their communicative interaction from the start of the visit. According to these authors, there are three main possible scenarios: (a) the doctor’s beginning, which was the most frequent case in the pre-computer era; (b) the patient’s beginning, in which the patient defines the interactive flow of the visit based on his/her agenda; (c) the computer’s beginning, in which the computer shapes the visit from the first minute. In this latter case, the doctor prioritizes the computer over the patient and uses technology to manage and guide the flow of communication. Greatbatch et al. (34, 35) showed how both the doctor and the patient align their activities with computer activity. Patients synchronize their gaze and speech with the doctor’s ongoing activity, for example, avoiding interrupting the physician’s typing, which is unlike what happens when the doctor writes by hand.

On the other hand, the use of computers in medical consultations has been found to cause longer physician response time. This is due to the many tasks the physician must manage simultaneously, including dividing attention between the patient and the computer, coping with abrupt topic changes to obtain the necessary information from the technology, and providing at least minimal answers to patients’ questions. Additionally, the use of computers has been linked to a loss of eye contact and less psychosocial information gathering during consultations (32, 36).

Newman et al. (37) utilized conversation analysis to investigate the communicative behavior of participants and their use of computer and paper-based artifacts in videotaped general practice visits. They found that when doctors used computers (instead of pen-and-paper), pauses were more likely to exceed 10 s and patients often broke the silence with distracting questions. Doctors also found it challenging to maintain the conversation’s topic after these long “computer-based” pauses.

Also Margalit et al. (38), who examined the time spent by doctors and patients on various activities during consultations, found a significant negative correlation between time spent on the computer and the number of questions asked by patients. Furthermore, the more a doctor uses the computer during consultations (in the so-called high-use computer visits), the longer the visit tends to be, as shown by McGrath et al. (39).

Greatbatch et al. (34, 35) conducted a longitudinal study of general practitioners visits, following the dismissal of paper-based systems and the progressive introduction of digital technologies. Through a micro-analysis of the video-recorded consultations, authors described how technology use impact on both the phisician’s and the patient’s communicative practices. Specifically, the increased reliance on computational tasks during medical visits had a significant impact on doctors’ communication behavior. They focused more on using the computer and less on direct interaction with the patient. This resulted in longer pauses as they waited for screen changes, sudden changes in topics to gather necessary information from the system, and shorter and less detailed responses to patient inquiries. Patients also had to adjust their communication to match the doctor’s computer-related activities, which was difficult as they often could not see the computer screen.

Overall, these studies strongly suggest that technology has emerged as a dominant presence in medical visits, demanding substantial time and attention from doctors and patients alike. Unfortunately, this heightened reliance on technology has the potential to hinder doctors’ ability to effectively engage in patient-centered communication practices.

## The pros and cons of using electronic medical records

Seminal ethnomethodological science and technology studies have shed light on the intricate relationship between medical documents and the professional practice of doctors (40, 41), revealing that the process of digitizing medical records is not simply a transfer from paper to screen. Instead, it should be seen as a mediator of a different representation of medical work (and of the patient’s body), rather than an “innocuous storage device” (42), p. 532. For this reason, the introduction of electronic medical records (EMRs) into medical practice has also significantly impacted the doctor-patient communication (43, 44).

Some authors have reported that physicians exhibit potentially negative communicative practices when using EMRs, such as interrupting talk from both patient and doctor, increased gaze shifting, and a low frequency of screen sharing with patients (45, 46). Swinglehurst et al. (47) conducted a linguistic ethnographic study on general practices and discovered that doctors often experience a “dilemma of attention” when balancing the immediate interpersonal interaction with the institutional requirements imposed by the electronic medical record (EMR) system. With the EMR open on the screen, doctors may be interrupted by prompts and alerts that demand their attention while scrolling through different parts of the record or entering information. These tasks frequently disrupt the flow of conversation and coordination between the doctor and patient.

Margalit et al. (38) found that physicians spend approximately a quarter of the visit time looking at the EMR on the computer screen, which adversely affects their engagement in psychosocial questioning and emotional responsiveness. Authors noted a negative correlation between the time spent looking at the screen and the time spent asking questions to patients. As the time spent typing on the physician’s computer increases, physicians tend to ask more closed-ended questions, and patients provide less detailed information about their health status. Furthermore, Margalit et al. (38) calculated a patient-centered communication score that demonstrated how physicians using computers and EMRs can negatively affect patient-centered practice by reducing interaction and dialogue, eye contact with the patient and affective behaviors.

Moreover, during medical encounters involving EMRs, the level of non-verbal communication is lower, and the visit duration is longer. These findings are consistent with other studies (36, 48, 49), which also reported that the increased visit duration is not utilized for speaking with patients, but for looking at the screen and typing on the keyboard. This additional time is filled with silence, minimal verbal engagement, and a reduction in overall interaction with patients.

Also Detmer and Gettinger (50) emphasized that the use of EMRs diminishes the amount of “clinical time” allocated to patient interaction and addressing care-related matters, as more time is spent on administrative and financial data entry into the computer.

They propose using a creative and innovative approach, including technological new solutions, to overcome the deterioration in the patient-clinical relationship caused by the current usage of EMRs. For example, these administrative tasks should be eliminated or automated using voice recognition or AI technology, freeing up time and space to facilitate more effective and satisfying patient-centered communication and care.

Interesting insights for addressing the challenges in the clinical relationship created by the use of EMRs also come from studies that have highlighted the positive impact of pen-and-paper literacy practices on patient-centered communication (PCC).

Sterponi et al. (51) adopting a conversational and multimodal approach, deeply analyze the “endangered literacy practice” in a corpus of video-recorded oncological visits. Doctors who use pen and paper practices follow a “interactional pattern,” enabling them to write and perform multiple complex activities (such as his diagnostic reasoning and the therapeutic decision making process) while simultaneously engaging with patients. Moreover these “slow” pen and paper practice enable doctors to share explanations of the disease and treatment options, promoting patient’ s active participation, engagement and understanding see also (52, 53).

These analog “slow” practices effectively support a patient-centered communication approach. Therefore, it is crucial to preserve and integrate them, rather than losing them, in the widespread transition to digital in order to achieve and sustain rich and meaningful patient-centered communication practices. For example, some authors (54, 55) demonstrate that sharing the screen with the patient is an effective way to support patient-centered communication practices: displaying test results, examples, and graphs on the computer screen allows for shared discussion with patients about their disease and available treatment options. This “open” approach enhances patient engagement and socialization, thereby improving their comprehension of the illness and treatment. And also explaining to the patient what the clinician is doing on the computer, explicitly accounting for any temporary suspension of the interaction, can be useful for fostering the patient’s active participation in the visit.

## Concluding remarks: some findings and implications

According to our literature review, what are the condition, resources and costraints for utilizing technologies to foster and enhance patient-centered communication during medical visits? We outline and analyze here three key considerations for orienting concrete medical practice.

**Figure 1:**
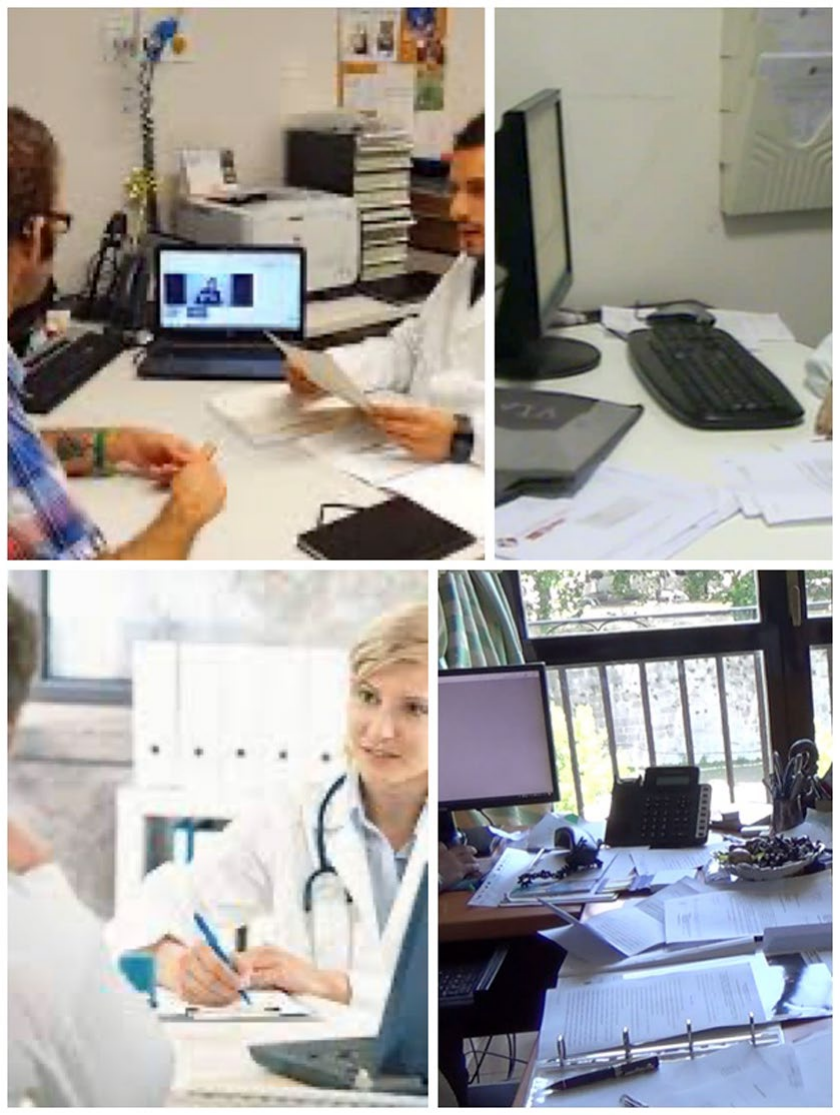
Heterogeneous digital and analog tools.

1. Promote a shared and transparent use of technology for the patient. The affordances of technologies do not depend on their technical or material characteristics but rather on the courses of action and communication they produce and support [cf. (56)]. It is crucial, therefore, to establish “good” configurations between technical aspects and socio-communicative aspects, which allow for the use of technology to enhance the empowered participation of the patient in medical visit activities and improve patient-centered communication practices (such as making the screen visible to share explanations of clinical exams or the choice between risks associated with different treatment options, or informing the patient about what is being done) [cf. (54)].
2. Integrate the affordances of analog technologies while incorporating the benefits of digital tools. The practices of interaction and communication between doctor and patient are mainly characterized by the simultaneous use of various heterogeneous digital and analog tools, which have been assembled over time in more or less coherent ways with the execution of medical activities (cf. Figure 1).

It is important, therefore, to implement a coordinated and integrated use of the different communicative affordances of these tools in relation to the activities to be carried out during the visit, in order to help patients become more competent and empowered participants in the interaction [cf. (51)].

1. Re-designing “creatively” also the technologies already in use (such as EMRs). By utilizing the new possibilities offered by recent technological and digital advancements, these systems can be re-designed to become patient-centered technologies, relieving physicians from routine and time-consuming administrative tasks that burden the clinical interaction with patients [cf. (50)]. These developments could improve the space and quality of doctor-patient communication, enhancing the quality of care and the satisfaction of all participants in the visits (physicians, patients, and caregivers).

## Author contributions

FM and FA conceived of the presented idea. FM developed the work and wrote the original draft and revised and edited the final version of the paper. FA revised and edited the article critically for final submission. CZ contributed to the final manuscript version and revised and edited the article critically for final submission. MF revised the final version of the article. All authors contributed to the article and approved the submitted version.

## Funding

This paper has been funded through the “Progetto per l’avvio della ricerca di tipo 2” - Sapienza University of Rome. Grant number: AR2221816467CB30.

## Conflict of interest

The authors declare that the research was conducted in the absence of any commercial or financial relationships that could be construed as a potential conflict of interest.

The handling editor AE declared a shared affiliation with the author(s) at the time of review.

## Publisher’s note

All claims expressed in this article are solely those of the authors and do not necessarily represent those of their affiliated organizations, or those of the publisher, the editors and the reviewers. Any product that may be evaluated in this article, or claim that may be made by its manufacturer, is not guaranteed or endorsed by the publisher.
